# Editorial: Unravelling Copper-Regulatory Systems and Copper-Affected Pathways in Cancer Cells to Improve Current Therapies

**DOI:** 10.3389/fmolb.2022.876902

**Published:** 2022-03-21

**Authors:** Xin Chen, Chiara Nardon, Q. Ping Dou

**Affiliations:** ^1^ Guangzhou Municipal and Guangdong Provincial Key Laboratory of Protein Modification and Degradation, Affiliated Cancer Hospital and Institute of Guangzhou Medical University, School of Basic Medical Sciences, Guangzhou Medical University, Guangzhou, China; ^2^ Department of Biotechnology, University of Verona, Verona, Italy; ^3^ Barbara Ann Karmanos Cancer Institute and Departments of Oncology, Pharmacology and Pathology, School of Medicine, Wayne State University, Detroit, MI, United States

**Keywords:** copper, cancer, metabolism, cell death, proteasome, ubiquitination

Copper is an essential bio-inorganic element that plays important roles in many processes in vertebrates. Elevated serum and tumor copper levels have been reported in numerous solid and hematologic malignancies. Therefore, targeting copper homeostasis has emerged as a favorable strategy in cancer treatment. On the one hand, copper chelation has demonstrated efficacy in inhibition of tumor growth and angiogenesis; on the other hand, copper supplementation by using copper complexes/ionophores can cause death in cancer cells. Thus, it is crucial to identify and characterize underlying mechanisms of copper-dependent signaling and copper-mediated cytotoxicity in cancer cells.

This Research Topic of **Frontiers in Molecular Biosciences,** entitled “*Unravelling Copper-Regulatory Systems and Copper-Affected Pathways in Cancer Cells to Improve Current Therapies*”, contains four (4) reviews focusing on copper-related pathways, copper-targeting agents and cancer. These articles have reviewed general mechanisms of copper metabolism in cancer cells, and summarized the action mechanisms of copper complexes/ionophores and copper-binding agents, highlighting the anticancer potential of copper-based strategies.

L. M. Ruiz etal. discussed recent perspectives on the role of copper in mitochondrial function and metabolism of malignant cells. Mitochondria play an essential role in cell metabolism. In mitochondria, copper is preferentially delivered to cuproenzymes, such as the respiratory chain complex IV (COX) and the antioxidant enzyme superoxide dismutase 1 (SOD1). COX is a cuproenzyme involved in the mitochondrial respiratory chain by catalyzing the reduction of molecular oxygen to water. The important role of copper in COX-mediated ATP generation suggests that this bio-metal center is fundamental for sustaining vital processes. SOD1 is a critical copper/zinc-containing antioxidant enzyme localized predominantly in the cytosol, and it is also present in the intermembrane space of mitochondria. The function of SOD1 in the mitochondria is still unknown, but it might be capable of reducing ROS (released from the respiratory chain) in the intermembrane space of mitochondria. Moreover, intracellular copper has an impact on cancer cell proliferation or differentiation by altering mitochondrial function or inducing metabolic reprogramming. A better understanding of the copper role in mitochondrial function and metabolism may open a novel avenue in cancer prevention and treatment.

Chen et al. extensively reviewed several copper complexes targeting the ubiquitin-proteasome system (UPS) in cancer cells. In the UPS, ubiquitination leads to the covalent attachment of ubiquitin to target proteins, followed by degradation *via* the proteasome, whereas the deubiquitinase enzyme (DUB) reverses protein ubiquitination and rescues the target protein from proteasomal degradation. As an additional machinery required for the UPS, the Cdc48–Ufd1–Npl4 complex extracts polyubiquitinated proteins from membranes or macromolecular complexes, and presents them as substrates to the 26S proteasome. Cancer cells rely on the production of multiple proteins to promote cell survival and proliferation, thus being highly sensitive to the inhibition of the UPS. Several copper complexes (*e.g*., CuET, CuHQ, CuCQ, CuPDTC, CuPT, and CuHK) have been proposed as potent anticancer agents by targeting one or more components of the UPS machinery, such as proteasome, DUB and NPL4 complex. The current challenge is to improve target selectivity of copper complexes towards the UPS.

The next comprehensive review by Kannappan et al. focuses on the scientific rationale, molecular targets, and mechanisms of disulfiram and its analogs in oncological therapy. Disulfiram has been approved by the U.S. Food and Drug Administration for the treatment of alcoholism. The metabolite of disulfiram, diethyldithiocarbamate (DDC), can bind copper *in situ* to form a complex with a 1:2 metal-to-ligand stoichiometry ([Cu(DDC)_2_]), which induces copper-dependent cell death (cuproptosis) or apoptosis in cancer cells. Several mechanisms are involved in the disulfiram action. First, the reaction of disulfiram or DDC with copper generates ROS that promote cancer cell death. Secondly, disulfiram/copper and [Cu(DDC)_2_] inhibit the proteasome activity in many types of cancer cells *in vitro* and *in vivo*. Thirdly, [Cu(DDC)_2_] targets the NPL4 pathway upstream of proteasome, resulting in accumulation of ubiquitinated proteins and cell death in cancer cells. Fourthly, disulfiram or disulfiram-copper complex effectively suppressed tumour progression by inhibiting the aldehyde dehydrogenase isoform 1 (ALDH1) activity in cancer stem cells. However, disulfiram showed short half-life and low bioavailability, limiting its application in the clinical setting. Therefore, the development of controlled-release nanoplatforms will help the successful clinical translation of disulfiram.

Finally, Oliveri reviewed the anticancer activity of copper ionophores and the potential strategies for further improving their selectivity towards cancer cells. Copper ionophores are copper-coordinating compounds that can transport copper into cells, increasing its intracellular bioavailability. Many copper ionophores are endowed with antitumor activity, including dithiocarbamates, bis(thiosemicarbazone) ligands, elesclomol, and 8-hydroxyquinolines. ROS induction is one of the primary mechanisms responsible for the antiblastic action of these copper ionophores. Due to high levels of intracellular ROS, cancer cells are very vulnerable to ROS induction. Thus, use of copper ionophores would be a selective anti-tumor therapeutic strategy. Several efforts have been made to improve the selectivity of copper ionophores, including the design of proionophores and the application of nanotechnology for drug delivery. Regarding the administration of metal-binding compounds, careful evaluation of the side effects and safety of copper ionophores is still needed prior to clinical trials.

Taken together, we anticipate that these timely reviews will provide inspiration for antitumor drug development. Although remarkable progress has been made, this field still faces certain challenges. The development of low-toxicity and high-efficacy copper complexes as anticancer drugs is greatly desired. With the rapid advancement of the sequencing and genome-editing technologies, high-throughput methods will provide novel insights into the role of copper in neoplasia and the exact targets or specific anti-tumor mechanisms of new copper-based agents. The use of patient-derived organoids is warranted in the future, likely contributing significantly to the development of selective copper-affecting or copper-based compounds as innovative chemotherapeutics.

The guest editors would like to thank all the authors - who are experts in the abovementioned research areas - for their remarkable contributions.

